# Efficacy of osteopathic manipulation as an adjunctive treatment for hospitalized patients with pneumonia: a randomized controlled trial

**DOI:** 10.1186/1750-4732-4-2

**Published:** 2010-03-19

**Authors:** Donald R Noll, Brian F Degenhardt, Thomas F Morley, Francis X Blais, Kari A Hortos, Kendi Hensel, Jane C Johnson, David J Pasta, Scott T Stoll

**Affiliations:** 1 New Jersey Institute of Successful Aging, University of Medicine and Dentistry - School of Osteopathic Medicine, Stratford, New Jersey, USA; 2 AT Still Research Institute, AT Still University, Kirksville, Missouri, USA; 3 Department of Medicine, University of Medicine and Dentistry of New Jersey - School of Osteopathic Medicine, Stratford, New Jersey, USA; 4 Department of Internal Medicine, Galion Community Hospital, Galion, Ohio, USA; 5 Michigan State University - College of Osteopathic Medicine Macomb University Center, Clinton Township, Michigan, USA; 6 Department of Osteopathic Manipulative Medicine, University of North Texas Health Science Center at Fort Worth - Texas College of Osteopathic Medicine, Fort Worth, Texas, USA; 7 ICON Clinical Research, San Francisco, California, USA; 8 Stoll Neurodiagnostics, PA, Fort Worth, Texas, USA

## Abstract

**Background:**

The Multicenter Osteopathic Pneumonia Study in the Elderly (MOPSE) is a registered, double-blinded, randomized, controlled trial designed to assess the efficacy of osteopathic manipulative treatment (OMT) as an adjunctive treatment in elderly patients with pneumonia.

**Methods:**

406 subjects aged ≥ 50 years hospitalized with pneumonia at 7 community hospitals were randomized using concealed allocation to conventional care only (CCO), light-touch treatment (LT), or OMT groups. All subjects received conventional treatment for pneumonia. OMT and LT groups received group-specific protocols for 15 minutes, twice daily until discharge, cessation of antibiotics, respiratory failure, death, or withdrawal from the study. The primary outcomes were hospital length of stay (LOS), time to clinical stability, and a symptomatic and functional recovery score.

**Results:**

Intention-to-treat (ITT) analysis (n = 387) found no significant differences between groups. Per-protocol (PP) analysis (n = 318) found a significant difference between groups (P = 0.01) in LOS. Multiple comparisons indicated a reduction in median LOS (95% confidence interval) for the OMT group (3.5 [3.2-4.0] days) versus the CCO group (4.5 [3.9-4.9] days), but not versus the LT group (3.9 [3.5-4.8] days). Secondary outcomes of duration of intravenous antibiotics and treatment endpoint were also significantly different between groups (P = 0.05 and 0.006, respectively). Duration of intravenous antibiotics and death or respiratory failure were lower for the OMT group versus the CCO group, but not versus the LT group.

**Conclusions:**

ITT analysis found no differences between groups. PP analysis found significant reductions in LOS, duration of intravenous antibiotics, and respiratory failure or death when OMT was compared to CCO. Given the prevalence of pneumonia, adjunctive OMT merits further study.

## Background

Pneumonia is the fourth most common hospital discharge diagnosis in the US with a mean (SE) length of stay (LOS) of 5.1 (0.1) days [1]. The elderly are a vulnerable population: the majority of pneumonia-related hospital admissions occur in persons 60 years and older, and the elderly have a longer mean LOS, higher severity of illness, and greater mortality than younger age groups [2-5]. Adjunctive nonpharmacologic treatments for pneumonia may enhance conventional antibiotic therapy. Chest physiotherapy, early mobilization, and continuous lateral rotational therapy have been investigated with mixed results [6-11]. Osteopathic manipulative treatment (OMT) is a nonpharmacologic manual therapy developed in the late nineteenth century before the use of antibiotics. OMT includes a number of manipulative techniques intended to enhance host defenses and physiologic function [12-16]. Many of these techniques, such as rib raising, doming the diaphragm, and the thoracic lymphatic pump, were specifically developed to treat pneumonia [15]. Two small randomized controlled trials of OMT for pneumonia suggest OMT may reduce LOS in the elderly [17,18].

The Multicenter Osteopathic Pneumonia Study in the Elderly (MOPSE) was designed to further evaluate the efficacy of OMT. To better understand the potential therapeutic effect of the interaction between participants and providers, MOPSE included sham treatment and conventional care only control arms. The primary hypotheses were that OMT would reduce LOS, time to clinical stability [19], and a symptomatic and functional recovery score [20] in elderly patients hospitalized with pneumonia compared to light touch sham and conventional care only control groups. Analysis of secondary and safety-related outcomes was also performed.

## Methods

MOPSE is a registered (http://www.clinicaltrials.gov, NCT00258661), double-blinded, randomized, controlled trial conducted from March 2004 to April 2007 with a 60-day post-admission follow-up period.

**Figure 1:**
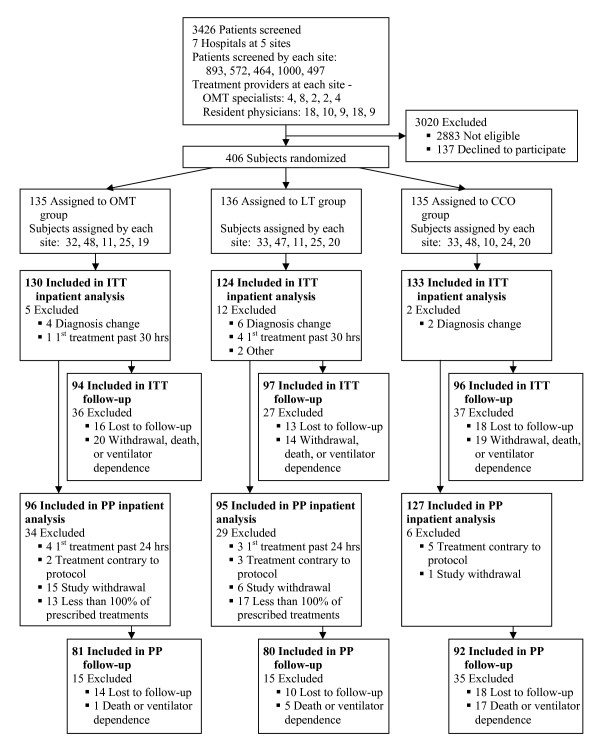
**Flow diagram of the Multicenter Osteopathic Pneumonia Study in the Elderly**. ITT = intention-to-treat, PP = per-protocol, OMT = osteopathic manipulative treatment, LT = light-touch treatment, CCO = conventional care only. Data on each site are presented in the following order: Michigan, Missouri, New Jersey, Ohio, and Texas.

Subjects aged ≥ 60 years newly hospitalized with pneumonia were recruited for MOPSE. Ten months into the study the age criterion was lowered to ≥ 50 years to improve recruitment. Eligibility criteria included a new pulmonary infiltrate on chest x-ray and at least two of the following: new or increased cough, fever ≥ 38°C, pleuritic chest pain, new physical findings on chest examination, respiratory rate ≥ 25 breaths/min, deteriorating mental or functional status, or white blood cell count (WBC) > 12,000 cells/mm^3^. Exclusion criteria were nosocomial pneumonia, lung abscess, advancing pulmonary fibrosis, bronchiectasis, pulmonary tuberculosis, lung cancer, metastatic malignancy, uncontrolled metabolic bone diseases, current rib or vertebral fracture, prior pathologic fracture, previous study participation, or respiratory failure. See Figure 1 for the MOPSE flow diagram.

Subjects were recruited at seven community hospitals in one rural, two suburban, and two urban US communities. The respective institutional review boards approved the study protocol, and all participants gave informed consent. An independent data and safety monitoring board provided oversight for MOPSE.

Subjects were enrolled and randomized into three groups: conventional care only (CCO, standard care control), light-touch treatment (LT, sham control), or osteopathic manipulative treatment (OMT). All subjects received conventional treatment for pneumonia directed by their attending physicians. Subjects were stratified by study site and randomized from a computer-generated list using block sizes of 3 or 6. Allocation was concealed with opaque, sealed envelopes which were opened after enrollment. Subjects, personnel responsible for collecting data, attending physicians, nurses, and house staff caring for subjects were blinded to group assignment. Only the physicians giving the study treatments were unblinded to group assignment.

Participants randomized to the OMT or LT groups received protocol treatments for 15 minutes, twice daily (≥ 6 hours apart) beginning within 24 hours of admission and continuing until hospital discharge, cessation of antibiotic therapy for pneumonia, respiratory failure (ventilator dependent), death, or study withdrawal. All subjects were treated while supine in bed. The manipulation techniques of the OMT protocol were administered in the following sequence: thoracolumbar soft tissue, rib raising, doming of the diaphragm myofascial release, cervical spine soft tissue, suboccipital decompression, thoracic inlet myofascial release, thoracic lymphatic pump, and pedal lymphatic pump [15]. Soft tissue technique consists of massage, stretching, kneading, and direct inhibitory pressure to relax the musculature. Rib raising articulates each rib for the purpose of improving rib cage motion and theoretically stimulates the sympathetic chain ganglia. Myofascial release is a method for reducing tissue tension. Doming the diaphragm and thoracic inlet myofascial release techniques are used to improve diaphragmatic movement and lymphatic drainage. Suboccipital decompression involves traction at the base of the skull, which is considered to release restrictions around the vagus nerves, theoretically improving nerve function. The thoracic lymphatic pump with activation combines rhythmical compressions to the chest wall and the rapid removal of the hands from the chest wall during deep inhalation with the intention of enhancing lymphatic circulation and triggering a sudden expansion of airways and alveoli. The pedal lymphatic pump gently rocks the patient in a superior-inferior rhythmical motion while supine, to theoretically enhance lymphatic circulation.

Non-thrust techniques were used to treat areas unaddressed by the above techniques, limited to ≤ 5 minutes of the 15-minute session. The LT protocol, designed as a sham control treatment, applied light touch to the same body regions, in the same sequence, and for the same duration as the OMT protocol. A more detailed description of the OMT and LT protocols has been published [15].

Twenty osteopathic neuromusculoskeletal (OMT) specialists and 64 resident physicians from 12 specialties administered the protocols. An OMT specialist at each site administered one of the first two OMT or LT treatments and 3 treatments per week thereafter. Using a standardized patient, treatment skills and protocol adherence were evaluated by the principal investigator and an OMT specialist at 9 training sessions at each site during the study. A pressure mapping system (Sensor Products LLC, East Hanover, NJ) was used to standardize three OMT techniques: rib raising, suboccipital decompression, and thoracic lymphatic pump. Presentation of the pressure mapping data is beyond the scope of this manuscript.

Primary outcomes were LOS, time to clinical stability [19], and a symptomatic and functional recovery score [20]. LOS was defined by the date and time of the admission and discharge orders. Based on data recorded daily, time to clinical stability was defined as the hospital calendar day when all seven clinical parameters first met criteria for stability (ie, lowest systolic blood pressure ≥ 90 mmHg, highest heart rate ≤ 100 beats/min, highest respiratory rate ≤ 24 breaths/min, highest temperature ≤ 38°C, lowest oxygen saturation ≥ 90%, ability to eat food by mouth or by a feeding tube, and mental status back to pre-pneumonia baseline) [19]. The symptomatic and functional recovery score was calculated from a pneumonia-specific, validated questionnaire addressing five symptoms: cough, dyspnea, sputum production, pleuritic chest pain, and fatigue [20]. Higher scores indicate more symptoms. This questionnaire was administered at admission and via telephone on post-admission days 14, 30, and 60.

Secondary outcomes were duration of intravenous and oral antibiotics; treatment endpoint, including death and respiratory failure; 60-day hospital readmission rate; highest daily temperature; highest daily respiratory rate; and WBC. Baseline severity of illness was assessed using the pneumonia severity index [21]. Adverse events, whether related to the study treatments or not, were monitored daily while subjects were in the hospital. Treatment side effects were evaluated 24 hours post-discharge; subjects were asked to report the severity and character of muscle soreness, worsening of breathing, or other side effects. The success of subject blinding was assessed via questionnaire within 24 hours of hospital discharge.

Using pilot data [18] to estimate the median LOS for the OMT and CCO groups, a log-rank test for survival has 80% power to detect a difference in median LOS of 6 versus 9 days when the sample size in each group is 96 subjects discharged from the hospital. MOPSE was designed to enroll 120 subjects per group with censored values (death, respiratory failure, or study withdrawal) estimated at ≤ 20% of the subjects.

Data were entered in duplicate at the study site and the central coordinating center, and checked for agreement. Data were analyzed by intention-to-treat (ITT) analysis and by per-protocol (PP) analysis of subjects receiving 100% of prescribed treatments. Data were analyzed for all subjects (≥ 50 years) and then separately for subjects who met the original age criterion (≥ 60 years). To test the hypotheses regarding group differences, we used three statistical analysis methods that stratify by study site to account for clustering. Groups were compared on LOS, time to clinical stability, and duration of in-hospital antibiotics using stratified Cox proportional hazards models in order to include incomplete data from subjects who withdrew from the study, died, or were placed on a ventilator. Hazard ratios for LOS greater than 1 correspond to earlier discharge from the hospital for the treatment group compared to the control group. Differences in the three groups on continuous outcome measures were assessed using general linear mixed-effects models with subjects and study sites treated as random effects and group assignment, time, and the interaction of group and time as fixed effects. The Cochran-Mantel-Haenszel test for general association was used to analyze categorical outcome measures and compare the incidence of adverse events and serious adverse events between the groups. P values ≤ 0.05 were considered statistically significant. The statistical analyses were performed using SAS^© ^version 9.1 (SAS Institute, Inc., Cary, NC).

## Results

### Primary and secondary outcomes

**Figure 2:**
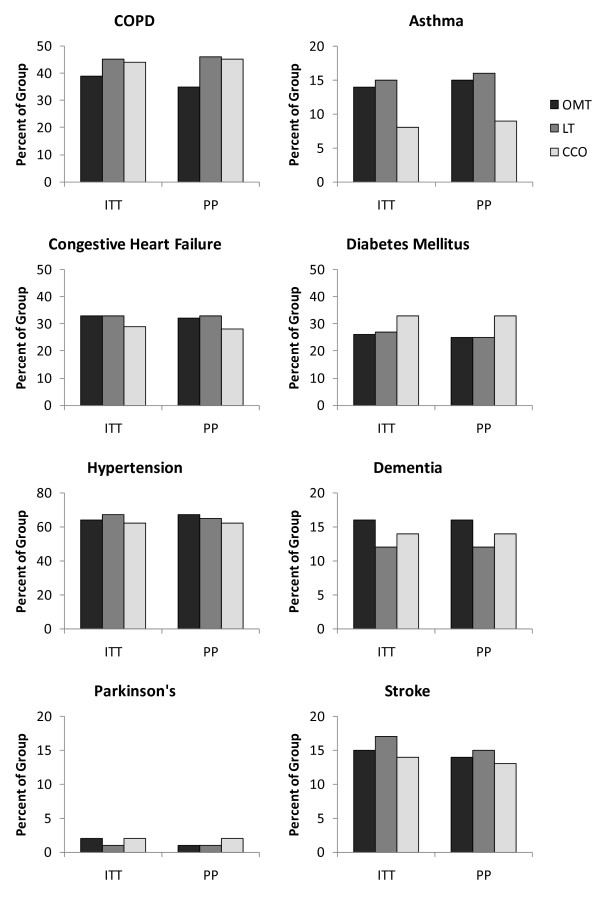
**Comparison between intention-to-treat and per-protocol analyses on MOPSE subject comorbidities**. ITT = intention-to-treat, PP = per-protocol, OMT = osteopathic manipulative treatment, LT = light-touch treatment, CCO = conventional care only, COPD = chronic obstructive pulmonary disease.

**Figure 3:**
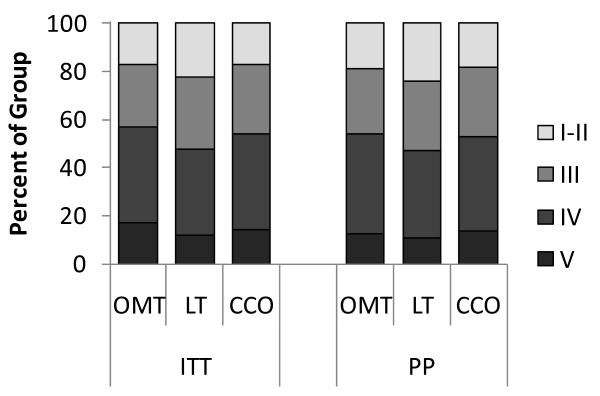
**Comparison between intention-to-treat and per-protocol analyses on MOPSE subject pneumonia severity**. ITT = intention-to-treat, PP = per-protocol, OMT = osteopathic manipulative treatment, LT = light-touch treatment, CCO = conventional care only.

A total of 406 subjects were enrolled and randomized. The final sample size for the ITT analysis was 387 subjects; 174 males (45%), 351 Caucasian (91%), mean age (SD) 73.7 (12.3) years. Although the selection of antibiotic therapy was managed by each participant's attending physician, 84% of subjects received antibiotic treatment in accordance with the published practice guidelines in place during the time of the study [22] and 94% of subjects received antibiotic treatment in accordance with the 2007 practice guidelines [23]. There was no significant difference between the groups on compliance with antibiotic treatment guidelines (P = 0.24 and 0.32, respectively). Demographic and clinical measures, including comorbidities and pneumonia severity, were not significantly different between groups (Table 1, Figures 2 and 3) with two minor exceptions, aspiration risk (P = 0.05, LT>CCO) for ITT analysis and current alcohol use (P = 0.03, OMT<LT and CCO) for PP analysis.

**Table 1:** Baseline Characteristics of the MOPSE Subjects

		OMT Group (n ITT = 130) (n PP = 96)	LT Group (n ITT = 124) (n PP = 95)	CCO Group (n ITT = 133) (n PP = 127)
**Sex - no. (%) Male**				
ITT Analysis		59 (45)	55 (44)	60 (45)
PP Analysis		43 (45)	39 (41)	59 (46)
**Age (yrs) - mean (SD)**				
ITT Analysis		73.8 (11.8)	74.6 (12.5)	72.8 (12.6)
PP Analysis		74.4 (12.0)	74.9 (12.8)	72.3 (12.6)
**Race - no. (%) White**				
ITT Analysis		122 (94)	110 (89)	119 (89)
PP Analysis		91 (95)	84 (88)	114 (90)
**Body Mass Index (kg/m^2^) - mean (SD)**				
ITT Analysis		27.6 (8.5)	27.8 (8.9)	27.4 (8.1)
PP Analysis		27.5 (7.8)	26.4 (7.3)	27.3 (8.3)
**Smoking History - no. (%) Current/Former**				
ITT Analysis	Current	25 (19)	20 (16)	34 (26)
	Former	41 (32)	48 (39)	39 (29)
PP Analysis	Current	18 (19)	17 (18)	32 (25)
	Former	27 (28)	36 (38)	39 (31)
**Current Alcohol Use by Chart Review - no. (%)**				
ITT Analysis		11 (9)	17 (14)	20 (15)
PP Analysis		4 (4)	14 (15)	19 (15)
**Comorbidities - no. (%)**				
ITT Analysis	COPD	51 (39)	56 (45)	59 (44)
	Asthma	18 (14)	19 (15)	11 (8)
	CHF	43 (33)	41 (33)	39 (29)
	DM	34 (26)	33 (27)	44 (33)
	HTN	83 (64)	83 (67)	83 (62)
	Dementia	21 (16)	15 (12)	19 (14)
	Parkinson's	2 (2)	1 (1)	2 (2)
	Stroke	19 (15)	21 (17)	18 (14)
PP Analysis	COPD	36 (35)	44 (46)	57 (45)
	Asthma	15 (15)	15 (16)	11 (9)
	CHF	33 (32)	31 (33)	37 (28)
	DM	26 (25)	24 (25)	43 (33)
	HTN	68 (67)	62 (65)	80 (62)
	Dementia	16 (16)	11 (12)	18 (14)
	Parkinson's	1 (1)	1 (1)	2 (2)
	Stroke	15 (14)	14 (15)	17 (13)
**Origin of Pneumonia - no. (%) Community-acquired (vs. Nursing Home-acquired)**				
ITT Analysis		105 (81)	98 (79)	103 (77)
PP Analysis		79 (82)	75 (79)	99 (78)
**Aspiration Risk by Chart Review - no. (%)**				
ITT Analysis		8 (6)	13 (11)	4 (3)
PP Analysis		5 (5)	8 (9)	4 (3)
**Pneumonia Severity Index - no. (%)**				
ITT Analysis	Class I-II	22 (17)	28 (23)	23 (17)
	Class III	34 (26)	37 (30)	38 (29)
	Class IV	52 (40)	44 (35)	53 (40)
	Class V	22 (17)	15 (12)	19 (14)
PP Analysis	Class I-II	18 (19)	23 (24)	23 (18)
	Class III	26 (27)	27 (28)	37 (29)
	Class IV	40 (42)	35 (37)	50 (39)
	Class V	12 (13)	10 (11)	17 (13)

ITT = intention-to-treat, PP = per-protocol, OMT = osteopathic manipulative treatment, LT = light-touch treatment, CCO = conventional care only, COPD = chronic obstructive pulmonary disease, CHF = congestive heart failure, DM = diabetes mellitus, HTN = hypertension.

ITT analysis found no significant difference between the groups for any outcome (Table 2). PP analysis found a significant difference between groups (P = 0.01) in LOS. Multiple comparisons indicated a reduction in median LOS (95% confidence interval [CI]) for the OMT group (3.5 [3.2-4.0] days) versus the CCO group (4.5 [3.9-4.9] days), but not versus the LT group (3.9 [3.5-4.8] days). There was also a significant difference between groups (P = 0.05) in the duration of intravenous antibiotics. Multiple comparisons indicated a reduction in median duration of intravenous antibiotics (95% CI) of 3.0 (2.7-3.5) days in the OMT group versus 3.5 (3.2-3.9) days in the CCO group. There was a significant difference between the groups on treatment endpoint (P = 0.006). Multiple comparisons indicated the treatment endpoints of death and respiratory failure were less frequent in the OMT versus the CCO group. Other outcomes were not statistically different, except respiratory rate, which was slightly lower in the OMT group than the CCO group (20.7 vs. 21.7 breaths/min, P = 0.04).

**Table 2:** Comparison of Treatment Groups on Primary and Secondary MOPSE Outcomes

Primary Outcomes				
	**OMT**	**LT**	**CCO**	**p value^a^**
**Hospital Length of Stay (days)**				
ITT Analysis	(n = 130)	(n = 124)	(n = 133)	
mean (SD)	4.5 (2.7)	4.9 (2.7)	4.5 (2.6)	
median (95% CI)	3.9 (3.4-4.7)	4.5 (3.8-4.9)	4.3 (3.9-4.9)	0.53
PP Analysis	(n = 96)	(n = 95)	(n = 127)	
mean (SD)	4.0 (2.0)	4.4 (2.4)	4.5 (2.6)	
median (95% CI)	3.5 (3.2-4.0)	3.9 (3.5-4.8)	4.5 (3.9-4.9)	0.01 (OMT<CCO)
**Time to Clinical Stability (days)**				
ITT Analysis	(n = 121)	(n = 118)	(n = 130)	
mean (SD)	2.5 (1.6)	2.5 (1.4)	2.6 (1.6)	
median (95% CI)	2.0 (2.0-2.0)	2.0 (2.0-3.0)	2.0 (2.0-2.0)	0.97
PP Analysis	(n = 90)	(n = 90)	(n = 124)	
mean (SD)	2.3 (1.4)	2.5 (1.5)	2.6 (1.6)	
median (95% CI)	2.0 (2.0-2.0)	2.0 (2.0-2.0)	2.0 (2.0-2.0)	0.47
**Symptomatic and Functional RecoveryScore**				
ITT Analysis - mean (SD)	(n = 99)	(n = 102)	(n = 99)	
- Admission	11.9 (4.6)	11.6 (4.4)	10.6 (4.6)	
- 14-day	5.8 (4.5)	5.0 (4.0)	4.4 (3.7)	Interaction (GroupxTime)
- 30-day	4.1 (4.4)	4.2 (3.6)	4.4 (3.7)	p = 0.24
- 60-day	4.0 (4.7)	4.0 (4.1)	3.4 (3.6)	Group p = 0.47
PP Analysis - mean (SD)	(n = 80)	(n = 80)	(n = 91)	
- Admission	11.6 (4.5)	11.6 (4.4)	10.5 (4.6)	
- 14-day	5.1 (3.9)	4.6 (3.8)	4.1 (3.4)	Interaction (GroupxTime)
- 30-day	3.8 (4.3)	4.0 (3.8)	4.2 (3.7)	p = 0.24
- 60-day	3.6 (4.9)	3.9 (4.2)	3.2 (3.6)	Group p = 0.73

**Secondary Outcomes**				

**Duration of Intravenous Antibiotic Use(days)**				
ITT Analysis	(n = 130)	(n = 124)	(n = 133)	
mean (SD)	3.7 (2.6)	3.9 (2.5)	3.9 (2.5)	
median (95% CI)	3.3 (2.9-3.7)	3.7 (2.9-3.9)	3.5 (3.1-3.9)	0.44
PP Analysis	(n = 96)	(n = 95)	(n = 127)	
mean (SD)	3.4 (1.9)	3.7 (2.5)	3.9 (2.5)	
median (95% CI)	3.0 (2.7-3.5)	3.3 (2.7-3.8)	3.5 (3.2-3.9)	0.05 (OMT<CCO)
**Duration of Total (Intravenous+Oral) Antibiotic Use (days)**				
ITT Analysis	(n = 130)	(n = 124)	(n = 133)	
mean (SD)	4.2 (2.7)	4.6 (2.5)	4.5 (2.5)	
median (95% CI)	3.6 (3.2-4.3)	4.1 (3.7-4.8)	4.0 (3.6-4.8)	0.43
PP Analysis	(n = 96)	(n = 95)	(n = 127)	
mean (SD)	4.0 (2.0)	4.4 (2.4)	4.5 (2.5)	
median (95% CI)	3.5 (3.0-3.9)	3.7 (3.1-4.6)	4.0 (3.6-4.8)	0.08
**Treatment Endpoint**				
ITT Analysis	(n = 124)	(n = 124)	(n = 132)	
n (%) Death	2 (2)	3 (3)	8 (6)	0.08
n (%) Respiratory Failure	4 (3)	4 (3)	10 (8)	
n (%) Discharge	118 (95)	117 (94)	114 (86)	
PP Analysis	(n = 96)	(n = 95)	(n = 127)	
n (%) Death	0 (0)	3 (3)	8 (6)	0.006^b^
n (%) Respiratory Failure	1 (1)	2 (2)	9 (7)	
n (%) Discharge	95 (99)	90 (95)	110 (87)	
**60-day Hospital ReadmissionRate**				
ITT Analysis	(n = 93)	(n = 96)	(n = 96)	
n (%) Readmission	16 (17)	20 (21)	21 (22)	0.64
PP Analysis	(n = 80)	(n = 79)	(n = 92)	
n (%) Readmission	9 (11)	16 (20)	19 (21)	0.16

ITT = intention-to-treat, PP = per-protocol, OMT = osteopathic manipulative treatment, LT = light-touch treatment, CCO = conventional care only, CI = confidence interval.

^a ^P values are for overall comparisons across three groups. Results from multiple comparisons are reported in parentheses.

^b ^Death and respiratory failure were less common in the OMT group compared to the CCO group.

**Figure 4:**
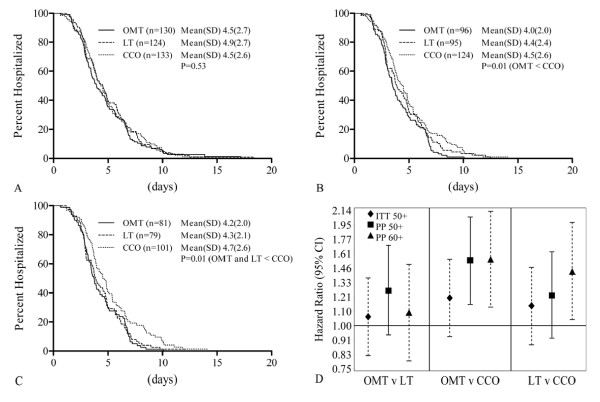
**Kaplan-Meier curves and hazard ratios for hospital length of stay comparing treatment groups**. OMT = osteopathic manipulative treatment, LT = light-touch treatment, CCO = conventional care only. A, Kaplan-Meier curves - intention-to-treat analysis on subjects aged 50 and above. B, Kaplan-Meier curves - per-protocol analysis on subjects aged 50 and above. C, Kaplan-Meier curves - per-protocol analysis on subjects aged 60 and above. D, Hazard ratios comparing treatment groups. Hazard ratios >1 correspond to an earlier discharge from the hospital for the first treatment group compared to the second. Calculated using intention-to-treat analysis on subjects aged 50 and above (ITT50+, diamond), per-protocol analysis on subjects aged 50 and above (PP50+, square), and per-protocol analysis on subjects aged 60 and above (PP60+, triangle).

Kaplan-Meier curves and hazard ratios (Figure 4) were calculated for the ITT analysis of subjects aged ≥ 50 (ITT50+), PP analysis of subjects aged ≥ 50 (PP50+), and PP analysis of subjects aged ≥ 60 (PP60+), who were included for consistency with the pilot data used in the power calculation. In Figure 4C, the PP60+ subgroup shows significantly lower LOS in both the OMT and LT groups relative to the CCO group (P = 0.01). Figure 4D, comparing OMT versus LT hazard ratios, shows no significant difference for any of the three subsets. OMT shows an advantage of shortened LOS over CCO in both PP50+ and PP60+, but not ITT50+. There was an advantage of shortened LOS for LT over CCO in the PP60+ analysis, but not ITT50+ or PP50+.

### Safety-related outcomes

**Figure 5:**
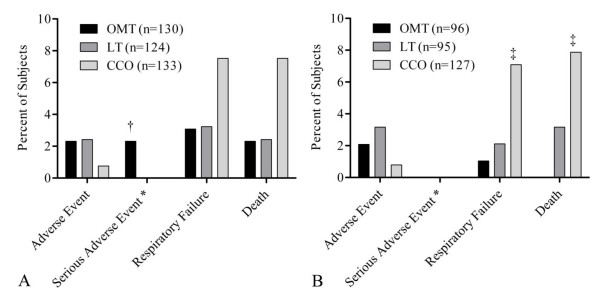
**Analysis of adverse events comparing treatment groups**. OMT = osteopathic manipulative treatment, LT = light-touch treatment, CCO = conventional care only. Subjects may be included in more than one category. For example, three subjects had respiratory failure as their treatment endpoint and subsequently died while still in the hospital. * Serious adverse event category excludes respiratory failure and death. A, Intention-to-treat analysis. † OMT significantly greater than LT and CCO, P < 0.05. B, Per-protocol analysis. ‡ OMT significantly less than CCO, P < 0.05.

Self-reported side effects were generally mild (posttreatment musculoskeletal soreness or pain) and significantly more common in the OMT group (P = 0.003; OMT 19/88 [22%], LT 6/86 [7%], CCO 5/76 [7%]) but resulted in the withdrawal of only one subject. Safety-related outcomes were determined by ITT and PP analyses (Figure 5). There were three serious adverse events in the OMT group, causing early withdrawal; none were OMT related per the data and safety monitoring board. PP analysis of serious adverse events showed significant differences between the groups on respiratory failure (P = 0.04) and death (P = 0.008). Multiple comparisons indicated that respiratory failure and death were higher in the CCO group (9/127 [7%] and 10/127 [8%], respectively) compared to the OMT group (1/96 [1%] and 0/96 [0%], respectively).

### Subject blinding

Subject blinding was assessed 24 hours post-discharge. By ITT analysis, many indicated they were "uncertain/not sure" of their group assignment (19/88 [22%] of OMT, 25/87 [29%] of LT, and 33/82 [40%] of CCO subjects). Approximately half correctly identified their group assignment (47/88 [53%] of OMT, 38/87 [44%] of LT, and 40/82 [49%] of CCO subjects). For those who believed they knew their group assignment, there was a significant relationship between their actual and perceived group assignment (P < 0.0001), with subjects more likely to identify their group assignment correctly than to guess they were in either of the other two groups. Of those who believed they knew their group assignment, more CCO subjects (40/49 [82%]) correctly identified their group assignment than OMT (47/69 [68%]) or LT (38/62 [61%]) subjects. PP analysis results were similar.

## Discussion

By ITT analysis, OMT did not improve outcomes. By PP analysis, OMT decreased LOS, duration of intravenous antibiotics, and the incidence of respiratory failure and death relative to CCO. Thus, if a subject received the OMT protocol as prescribed without missing any treatment sessions, there was significant benefit.

While the two studies conducted prior to MOPSE also suggest OMT reduces LOS [17,18], the mean LOS for all groups has been dramatically reduced since the early 1990s, from 2 weeks in the first pilot study to less than 5 days in the current study. These changes are consistent with national trends [1,24,25]. The current study was powered to detect a difference in median LOS of 6 versus 9 days, estimated using data from the single site study [18] when the LOS was 33%-100% longer than current standards. This LOS reduction limited the number of treatments given and the length of time to observe the potential impact of OMT and LT on the course of the disease. While all subjects in the current study continued to receive antibiotic therapy after hospital discharge, study treatments ended at hospital discharge. OMT may show greater efficacy when continued beyond acute care or when used in chronic care settings. In addition, a larger sample size may be needed to compensate for the reduced LOS.

Consistent with other studies [19,26], the time to clinical stability in the current study was two days, probably too short to identify between-group differences. The symptomatic and functional recovery score, a known measure of long-term recovery from pneumonia [20], did not show any between-group differences either. The 14% post-discharge loss to follow-up reduced the power and may have lessened the ability of this measure to detect between-group differences.

For the secondary outcome of treatment endpoint, death and respiratory failure were reduced for OMT relative to CCO by PP analysis. The LT group was intermediate between the other two groups and not significantly different from either. Because of the relatively low numbers involved, these findings should be interpreted with caution.

Significantly more patients receiving OMT experienced musculoskeletal soreness or pain sometime during the hospital stay. However, only one subject withdrew consequent to this side effect, and the severity was generally mild, suggesting OMT is reasonably tolerated even in this severely ill cohort.

Those making the clinical care decisions and collecting data and the subjects were blind to group assignment. The post-discharge survey suggests at least partial blinding since only half of the participants correctly identified their group assignment and 30% were unsure of their group assignment. It is surprising that only 49% of subjects in the CCO group correctly identified their group but not surprising that those in the CCO group who felt certain about group assignment more consistently identified their group. However, those in the OMT and LT groups who believed they knew their group assignment also more consistently identified their group. The impact of this partial blinding should be considered in future research.

A concern with PP analysis is that the exclusion of subjects may compromise the baseline similarity of the groups achieved by randomization, altering the risk-factor profile of the subjects within the groups [27,28]. In the current study, almost 18% of subjects (26% of OMT, 23% of LT, and 5% of CCO subjects) were excluded from the PP analysis due to missed treatments during hospitalization (43%), delayed initiation of treatments (10%), treatment contrary to protocol (15%), and study withdrawal (32%). As a result of these exclusions, subjects who met PP criteria in all three groups had significantly less severe pneumonia than those who were excluded (P = 0.005). However, the reasons for being excluded from PP analysis (protocol violation, study withdrawal, or receiving less than 100% of prescribed treatments) were not significantly related to pneumonia severity (P = 0.77). While more subjects were excluded from the OMT and LT groups than the CCO group for the PP analysis, the composition of the groups regarding demographics, comorbidities, and pneumonia severity was not significantly different with one minor exception for current alcohol use. This similarity suggests that the PP analysis sustained the between-group characteristics from the original randomization, minimizing the potential bias that can occur with PP analyses.

Another concern many have with PP analysis is whether the results represent real world conditions. While consistency of treatment is a valid concern and does influence the interpretation of data, improved compliance with the intended protocol can be achieved with current quality assurance mechanisms. In the current study, almost 70% of the subjects excluded from PP analysis were excluded for reasons that are mostly modifiable by healthcare providers (eg, initial treatment given more than 24 hours after admission, incorrect treatment given, missed treatment).

Alternatively, a concern with ITT analysis is that lack of adherence to the treatment protocol can result in underestimation of the impact of the treatment, particularly when the proportion of subjects who did not receive the full protocol treatment is sizable [28]. While this concern may be valid for the current study, the potential degree of underestimation was minimized due to the reduced LOS, which already limited the time for OMT efficacy.

Few studies have explored how osteopathic techniques might enhance host defenses. In rats, rhythmic mechanical pressure to body regions physically distant from the location of lymphatic formation enhances lymph uptake [29]. Knott et al [30] showed in a dog model that lymphatic pump techniques increased lymph flow through the thoracic duct, independent of cardiac variables. Hodge et al [31] demonstrated that abdominal lymphatic pump in dogs increased both thoracic duct flow and the leukocyte count in the lymph, thus increasing the mobilization of immune cells. In healthy male medical students, the lymphatic pump increased antibody response to pneumococcal polysaccharide [32]. However, two studies [33,34] in the elderly assessing the lymphatic pump on antibody response to the influenza vaccine showed no effect.

While the mechanisms are unclear, OMT may have a beneficial effect by enhancing mobilization as has been shown in early mobilization or kinetic bed therapy. Immobilization is a significant mortality-predicting characteristic in the elderly hospitalized with pneumonia [35]. Early mobilization, defined as out of bed at least 20 minutes daily from day one of hospitalization, reduced LOS in hospitalized patients with community-acquired pneumonia [9]. In patients requiring long-term assisted ventilation and in liver transplant recipients, kinetic or continuous lateral rotational bed therapy reduced the prevalence of pneumonia [8,11]. Unlike these therapies, OMT requires little equipment and can be given to patients unable to get out of bed.

MOPSE used a three-arm design to assess the potential therapeutic contributions of touch, attention, and expectation associated with manual therapies. If the LT outcomes were the same as OMT and both were better than CCO, then the primary benefits of OMT could be attributed to the therapeutic effect of LT. If the LT outcomes were the same as CCO and both were inferior to OMT, then the primary benefits of OMT could be attributed to therapeutic benefit of OMT. However, we found that the LT outcomes generally fell between OMT and CCO, not significantly different from either. This may indicate that the effect of OMT is partly due to whatever is therapeutic in LT and partly due to something inherent to OMT. One exception is the PP60+ subgroup, where both OMT and LT had significantly shorter LOS than CCO. It is interesting that the more elderly subgroup would be responsive to LT. These results highlight the need to compare the manipulative intervention to something more than a sham control since a two-arm study design may underestimate the overall value of the manipulative intervention if the sham control has some therapeutic value. The possible therapeutic components of a sham control (such as LT) are beyond the scope of this discussion.

MOPSE has the following limitations. The sample size was calculated to compare the OMT and CCO groups based on a longer LOS than the current national norm [1]; thus, the sample size may be too small to differentiate between the OMT and control groups. Although the MOPSE PP analysis found between-group differences, an ITT analysis more closely reflects real world conditions. OMT specialists administered only 3 of 8 treatments; the rest were administered by osteopathic resident physicians trained in the protocol. While results may improve with administration by OMT specialists, use of non-specialists trained in the protocol increases the generalizability of the MOPSE results. Although regular training sessions were conducted to standardize treatments among providers, there are no validated objective tools to measure the quality and uniformity of each care provider's technique.

## Conclusions

When OMT was administered in accordance with the protocol, reductions were seen in LOS, duration of intravenous antibiotics, and incidence of respiratory failure and death in the OMT group compared to the CCO group. Data suggest a reduced effect from LT compared to OMT in that LOS for LT was between CCO and OMT. These results suggest a potential role for OMT, and possibly for LT, to augment conventional antibiotic therapy in the treatment of pneumonia. This role may become more important with increasing antibiotic resistance, emerging pathogens, the aging global population, the cost of health care, and the likelihood of another influenza pandemic [36-38]. Since developing methods to address these issues is a major health care imperative, the effects of OMT and LT merit further investigation.

## Competing interests

DJP, as an employee of ICON Clinical Research, was paid by the Foundation for Osteopathic Health Services for statistical and analytical services. The other authors declare that they have no competing interests.

## Authors' contributions

DRN participated in the study concept and design, participated in the acquisition of the data, and helped draft the manuscript. BFD participated in the study concept and design, participated in the acquisition of the data, and helped draft the manuscript. TFM participated in the study concept and design, participated in the acquisition of the data, and helped draft the manuscript. FXB participated in the acquisition of the data and helped draft the manuscript. KAH participated in the study concept and design, participated in the acquisition of the data, and helped draft the manuscript. KH participated in the acquisition of the data and helped draft the manuscript. JCJ participated in the study concept and design, performed the statistical analyses, and helped draft the manuscript. DJP performed the statistical analyses and helped draft the manuscript. STS participated in the study concept and design, participated in the acquisition of the data, and helped draft the manuscript. All authors participated in the analysis and interpretation of the data, and read and approved the final manuscript.
